# Effects of Exercise on Cardiovascular and Metabolic Responses in Adults and Childhood Cancer Survivors: The Role of NETosis and Low-Grade Inflammation as a Novel Therapeutic Target—A Narrative Review

**DOI:** 10.3390/ijms262210843

**Published:** 2025-11-08

**Authors:** Rodrigo L. Castillo, Esteban G. Figueroa, Alejandro González-Candia, Andrea del Campo, Claudia Paris, Fernando Verdugo, Morin Lang, Carlos Cruz-Montecinos, Mauricio Quezada, Robert A. Pérez, Martín Armijo, Patricio Acevedo, Rodrigo Carrasco

**Affiliations:** 1 Departamento de Medicina Interna Oriente, Facultad de Medicina, Universidad de Chile, Santiago 7500922, Chile; martin.armijo@ug.uchile.cl (M.A.); patricio.acevedo.n@ug.uchile.cl (P.A.); 2 Unidad de Paciente Crítico, Hospital del Salvador, Santiago 7500922, Chile; 3 Escuela de Obstetricia, Facultad de Ciencias para el Cuidado de la Salud, Universidad San Sebastián, Santiago 7510602, Chile; esteban.figueroa@uss.cl; 4 Institute of Health Sciences, University of O’Higgins, Rancagua 2820000, Chile; alejandro.gonzalez@uoh.cl; 5 Facultad de Química y Farmacia, Pontificia Universidad Católica de Chile, Santiago 7820436, Chile; andrea.delcampo@uc.cl; 6 Unidad de Oncología, Hospital Calvo Mackenna, Santiago 7500539, Chile; cparis@calvomackenna.cl; 7 Laboratorio de Ecocardiografía, Servicio de Cardiologia, Hospital Salvador, Santiago 8320000, Chile; 8 Departamento de Kinesiología, Facultad de Medicina, Universidad de Chile, Santiago 8580453, Chile; morin.lang@uchile.cl (M.L.); carloscruz@uchile.cl (C.C.-M.); 9 Facultad de Medicina, Universidad Finis Terrae, Santiago 7501015, Chile; mauricioquezadac@gmail.com; 10 Facultad de Medicina, Universidad de los Andes, Santiago 7620086, Chile; raperez@miuandes.cl; 11 Department of Cardiology, University Heart Centre, University Hospital Zurich, University of Zurich, 8091 Zurich, Switzerland

**Keywords:** childhood and adult cancer survivors, exerkines, neutrophil extracellular traps, cardiometabolic risk, controlled exercise

## Abstract

Cancer survivors (CS) constitute an expanding population with underrecognized cardiometabolic risk. Despite substantial improvements in five-year survival rates, both childhood and adult survivors remain at high risk for premature morbidity and mortality. These risks are particularly pronounced following exposure to anthracyclines and/or chest radiotherapy, typically in a dose-dependent manner. In Chile, the establishment of the National Pediatric Antineoplastic Drug Program (PINDA) in 1998 marked a milestone in improving equitable access to high-quality pediatric oncology care through evidence-based treatment protocols across the public health system; the adult counterpart (PANDA) has developed diagnostic, treatment, and monitoring protocols for hematological neoplasms. Few prospective cohort or mechanistic studies have clarified risk stratification or surveillance strategies in survivor populations. The regulated, short-term activation of inflammation and innate immunity can be an adaptive and protective response to tissue injury, whereas persistent low-grade inflammation may trigger neutrophil extracellular traps formation (NETosis) and other maladaptive pathways that accelerate endothelial injury, thrombosis, and adverse cardiovascular remodeling. NETosis represents a putative immunomodulatory target for therapeutic immunomodulation in heart failure and maladaptive left ventricular remodeling in preclinical models. Concurrently, skeletal muscle-derived and hormonal mediators known as exerkines—together with increased NET activity—may modulate the pathophysiology of chronic cardiometabolic disease and contribute to cancer progression, particularly in the context of obesity, diabetes, and insulin resistance. Structured exercise is a promising non-pharmacological intervention that modulates inflammatory and metabolic pathways and may thereby help prevent non-communicable diseases, including cancer. We synthesize basic and clinical evidence to (1) define how cancer therapies promote low-grade inflammation and NETosis; (2) describe how exerkines and structured exercise influence cardiometabolic biology; and (3) evaluate exercise as a mechanistic and clinically pragmatic strategy to reduce long-term CVD risk in pediatric and adult CS.

## 1. Introduction

Childhood cancer survivors (CCS) represent a steadily expanding population worldwide, largely due to advances in multimodal oncologic therapies and supportive care. The U.S. National Cancer Institute defines cancer survivorship as encompassing “the health and life of a person with cancer after treatment until the end of life”, thereby underscoring the need for longitudinal care and surveillance beyond cancer remission [1]. Despite this remarkable therapeutic progress, survivorship remains frequently associated with substantial and underrecognized late effects, including an elevated burden of cardiometabolic risk. In the United States alone, there are nearly 500,000 survivors of childhood cancer, many of whom face lifelong health consequences, including premature cardiovascular disease (CVD), second primary neoplasms, and metabolic disorders [2]. Epidemiologic studies demonstrate that CCS experience a fifteen-fold increased risk of death from subsequent malignancies and a seven-fold increased risk of death from CVD [3], particularly among those exposed to anthracyclines or chest radiotherapy, in a dose-dependent manner [4]. Moreover, survivors frequently exhibit features of accelerate biological aging—including endothelial dysfunction, premature atherogenesis, and early frailty phenotypes—which are increasingly recognized as hallmarks of survivorship-related morbidity [5,6]. For this reason, elucidating the mechanisms that drive the early emergence of CVD risk factors in this population is of major clinical relevance.

Cancer survivors often experience declines in physical functioning and quality of life while simultaneously facing an increased risk of cancer recurrence and all-cause mortality compared with age-matched individuals without a history of cancer. Importantly, structured exercise has emerged as a safe and effective intervention for this population. The 2010 American College of Sports Medicine Roundtable concluded that cancer survivors can safely engage in appropriately prescribed exercise programs to improve physical fitness, restore physical functioning, enhance quality of life, and mitigate cancer-related fatigue [7]. In this context, emerging mechanistic evidence indicates that many of the benefits of controlled exercise are mediated through immuno-inflammatory pathways, particularly those involving skeletal muscle and visceral adipose tissue. These tissues act as key sites of immunometabolic regulation, and their response to exercise represents a critical target for long-term improvement in clinical outcomes among cancer survivors. In parallel, recent discoveries have highlighted the rapid release from activated immune-competent cells—NETosis, a process involving the release of neutrophil extracellular traps (NETs)—as an important pathophysiological mechanism. Elevations in circulating cell-free DNA (cfDNA) may reflect NET formation and/or active extracellular DNA release [8]. Interestingly, chronic high-intensity resistance training protocols, as well as long-duration endurance exercise, can induce persistent elevations in cfDNA levels, suggesting that exercise intensity modulates immune cell activation and systemic inflammatory responses that could lead to the slow, constant release of DNA [9].

Chronic low-grade inflammation—a shared hallmark of multiple non-communicable diseases (NCDs), including obesity and diabetes—contributes to endothelial dysfunction, accelerates cardiovascular risk factor progression, and may promote maladaptive cardiac remodeling in CS, particularly among pediatric and young adult populations [10]. This review synthesizes mechanistic and clinical evidence linking chronic inflammation to cardiometabolic risk in cancer survivors, with a special focus on exercise-induced modulation of skeletal muscle and adipose tissue as actionable therapeutic targets. By integrating molecular, physiological, and clinical perspectives, we aim to inform the development of optimized, multidisciplinary surveillance and intervention strategies designed to mitigate long-term cardiovascular and metabolic sequelae in this rapidly growing population.

## 2. Epidemiological Settings in Adults and Childhood Cancer Survivors

In Latin America and the Caribbean (LAC), CCS constitute a growing yet understudied population whose cumulative health burden underscores the pressing need for an integrated cardio-onco-rehabilitation (CORE) framework. Each year, >29,000 children and adolescents (0–19 years) are diagnosed with cancer, and ~10,000 die, making childhood cancer the second leading cause of death in this age group [11]. Although the regional average five-year survival rate approaches 55%, stark inequities persist between countries and subregions due to variations in health system capacity and access to care [11].

A recent multicenter survey of 135 CCS from 16 Latin American countries reported that >60% experience late mental-health effects, 59% had endocrine complications, and 42% reported cardiovascular sequelae, while fewer than one-third remained under regular medical follow-up [12]. These findings highlight a dual challenge: improving survival while addressing the scarcity of structured long-term surveillance and rehabilitation programs. Within this context, the CORE model offers a pragmatic, multidisciplinary strategy to mitigate cardio-metabolic and immunological risks, integrating exercise-based rehabilitation, biomarker-guided monitoring, and survivorship care tailored to regional health disparities [13].

Country-level data illustrate both the diversity and shared challenges of CCS across Latin America. In Brazil, an estimated 12,500 new pediatric and adolescent cancer cases were reported annually during the 2018–2019 biennium, with ~1300 cases concentrated in the southern region [14]. In 1962, Colombia established the first population-based cancer registry in the region, in the city of Cali [15], a milestone that prompted the creation of institutional registries across Argentina, Brazil, Chile, Ecuador, Honduras, and Peru, though most remain subnational in scope and offer limited utility for national policy-making. Currently, only Argentina and Chile maintain national pediatric cancer registries—Argentina’s Registro Oncopediátrico Hospitalario Argentino (ROHA), founded in 2000, remains the first and most comprehensive, enabling real-time data capture and nationwide coverage [16,17].

In Chile, national efforts exemplify the transition toward integrated pediatric oncology care. According to the Third Childhood Cancer Surveillance Report (2017–2019), leukemia remains the most frequently pediatric malignancy (~40%), and 5-year survival increased to 78.4% by 2023 [18]. The National Plan for Child and Adolescent Cancer Control—developed in collaboration with the Pan American Health Organization (PAHO)—has contributed to strengthening efforts in early diagnosis, continuity of care, and structured survivorship transition into adulthood. However, longitudinal follow-up remains limited (~10 years of registry data), and long-term morbidity in adulthood is still incompletely characterized.

Despite this progress, sustained multidisciplinary initiatives remain essential to close existing gaps [18]. Integrating CORE principles—clinical surveillance, exercise-based rehabilitation, and biomarker-guided cardiometabolic monitoring—offers a feasible path to optimize long-term outcomes and reduce cardiovascular and metabolic burden among CCS in Chile.

From a regional perspective, these national experiences illustrate broader patterns across Latin America, where disparities in access to care and variations in health-system capacity create heterogeneous survivorship trajectories. Therefore, the accelerated emergence of cardiometabolic risk factors, combined with a prolonged window of subclinical and asymptomatic disease, supports the implementation of proactive strategies for early detection and intervention, including lifestyle modifications and structured exercise programs. Integrating these approaches within longitudinal follow-up frameworks offers a promising opportunity to mitigate long-term cardiometabolic burden and optimize outcomes in this growing population of CCS.

## 3. Cardiovascular and Metabolic Status in Adults and Childhood Cancer Survivors

### 3.1. Adult Patients

Anthracyclines remain a cornerstone of therapy for leukemia and are also widely used in lymphoma, sarcomas, breast, uterine, and gastric cancers in Adult Cancer Survivors (ACS). However, their use is limited by dose-dependent cardiotoxicity [19]. The major risk factors for anthracycline-induced cardiomyopathy include cumulative dose, extremes of age, pre-existing cardiovascular disease, and concomitant chest irradiation; the highest incidence of heart failure occurs with cumulative exposures > 250 mg/m^2^ doxorubicin-equivalent doses [20]. As survival improves, morbidity from treatment-related cardiac damage—often irreversible and progressive—has become increasingly prominent. Preventive strategies such as limiting the cumulative dose, using alternative anthracycline formulations (e.g., pegylated liposomal doxorubicin), or administering cardioprotective agents (e.g., dexrazoxane) have shown clear benefits; however, their implementation in clinical practice remains inconsistent [21]. Notably, the interval between the emergence of cardiometabolic risk factors and the onset of overt cardiovascular disease provides a window for preventive, non-pharmacological interventions. Accordingly, optimal strategies must strike a balance between oncologic efficacy and long-term cardiovascular safety. This underscores the need for mechanistic studies and prospective clinical trials specifically addressing cardiotoxicity in ACS.

### 3.2. Pediatric Patients

Among children aged 0–14 years, leukemia (46.4%), central nervous system tumors (28.2%), and lymphomas (15.2%) dominate the global pediatric cancer spectrum, with this distribution consistently observed across world regions [22]. These malignancies, while increasingly treatable, expose survivors to treatment-related cardiotoxicity and metabolic disturbances, major determinants of long-term morbidity and mortality in both young adult and childhood cancer survivors (CCS). Indeed, cardiovascular disease is now recognized as the second leading cause of death after recurrent or secondary malignancies in CCS [23]. In this regard, CCS develop cardiometabolic risk factors earlier and more severely than peers, with elevated risk of premature cardiovascular disease (CVD) and multimorbidity. This excess risk is closely linked to prior exposure to anthracyclines, chest or mediastinal radiotherapy, hematopoietic stem cell transplantation, and novel targeted therapies, all of which contribute to premature vascular aging, myocardial dysfunction, and metabolic dysregulation [24].

Improved long-term follow-up of CCS is essential to mitigate cardiovascular risk during adulthood further. Several studies have demonstrated that subclinical cardiac, vascular, and lipid/apolipoprotein abnormalities are detectable in young ACS [25]. More specifically, impaired cardiac function and reduced carotid elasticity have been observed in survivors with greater treatment exposure, correlating with apolipoprotein abnormalities. Although these ultrasonographic, lipid, and apolipoprotein markers are well-established predictors of future CVD in other populations [26,27], their prognostic validity in ACS remains uncertain.

**Figure 1:**
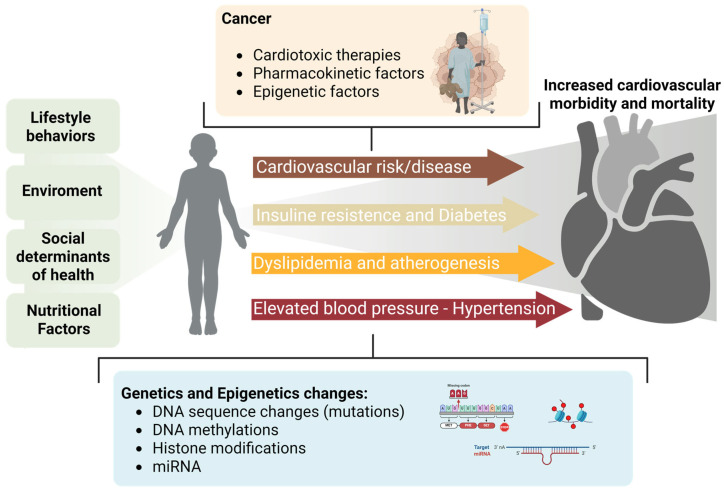
Proposed mechanisms linking cancer therapies, cardiometabolic risk factors, and cardiovascular disease in cancer survivors. Schematic representation of the interaction between clinical and laboratory risk factors in CS patients. CVDs related to cardiometabolic dysfunction arise primarily from the interplay between cancer, its therapies, and epigenetic alterations. These pathophysiological events contribute to cardiovascular morbidity and mortality in this population. Notably, several risk factors remain amenable to pharmacological modulation.

Given the long-term occurrence of CV events in this high-risk group, systematic monitoring of low-grade inflammation, oxidative stress, and tissue remodeling markers—integrated with functional cardiac and vascular assessments—could provide early mechanistic insights and enable timely, preventive interventions to halt disease progression before irreversible structural or functional damage occurs in CCS (Figure 1).

## 4. Monitoring of CV Risk Factors and Cardiac Function in Cancer Survivors

### 4.1. Surveillance of CV Risk Factors

Cardiovascular diseases represent a leading cause of long-term morbidity and mortality in cancer survivors. CVD and cancer share several traditional risk factors—such as obesity, smoking, and physical inactivity—yet cancer survivors are uniquely burdened by an increased prevalence of hypertension, dyslipidemia, and diabetes compared with the general population [28,29]. Data from the German CV-study demonstrated significantly elevated CV risks among CCS, including hypertension (RR 1.38; 95% CI 1.21–1.57), dyslipidemia (RR 1.26; 95% CI 1.12–1.42), and overt CVD (RR 1.89; 95% CI 1.34–2.66), with premature onset occurring approximately 6–8 years earlier than in the general population [29]. Despite this well-documented burden, modifiable risk factors remain frequently underdiagnosed and undertreated in cancer survivors [30], underscoring the critical need for heightened vigilance among healthcare providers and patients. Similarly, the ARIC (Atherosclerotic Risk in Communities) study confirmed that adult cancer survivors face an increased risk of incident CVD (HR 1.37; 95% CI 1.26–1.50), heart failure (HR 1.52; 95% CI 1.38–1.68), and stroke (HR 1.22; 95% CI 1.03–1.44), with traditional CV risk factors—such as smoking, diabetes and obesity—failing to fully explain this excess risk [31]. The combined effects of cancer-related physiological stress, treatment-induced toxicities, and adverse lifestyle changes during or after therapy appear to synergistically accelerate vascular aging and increase cardiac vulnerability, thereby contributing to the heightened burden of CV risk factors and overt CVD observed in cancer survivors [31,32]. Recent cardio-oncology guidelines advocate for systematic and lifelong surveillance of cardiovascular risk factors. According to the 2025 CORE (Cardio-Oncology Rehabilitation and Exercise) recommendations, cardiovascular risk assessment in cancer survivors should be structured and longitudinal, starting early after completion of oncologic therapy, and should include: (1) evaluation of traditional CV risk factors; (2) comprehensive lifestyle assessment; (3) measurement of metabolic and anthropometric parameters; and (4) laboratory evaluation of lipid profile, glucose metabolism, and—when available—emerging biomarkers. The early identification of high-risk individuals enables timely implementation of both pharmacological and non-pharmacological interventions—including exercise and nutrition programs—aimed at mitigating long-term CVD risk.

### 4.2. Cardiac Function Assessment

Cardiac function assessment is strongly recommended for high-risk cancer survivors to enable early detection of subclinical dysfunction. According to European Society of Cardiology/International Cardio-Oncology Society (ESC/ICOS) cardio-oncology guidelines, indications for cardiac assessment include: (1) patients classified as high-risk using the Heart Failure Association (HFA)-ICOS risk stratification tools; (2) survivors exposed to therapies with well-established long-term high-risk of cardiotoxicity, such as cumulative anthracycline doses ≥ 250 mg/m^2^ doxorubicin-equivalent, thoracic radiotherapy with a mean dose > 15 Gy, combined modalities at lower thresholds, or hematopoietic stem cell transplantation; (3) individuals who developed moderate-to-severe cancer therapy-related cardiotoxicity during treatment; and (4) patients presenting with new cardiovascular symptoms or abnormalities detected by transthoracic echocardiography (TTE) or cardiac biomarkers [33].

Among imaging modalities, TTE remains the most accessible and cost-effective tool for longitudinal surveillance, enabling evaluation of left ventricular (LV) systolic function—through LV ejection fraction (LVEF) and LV global longitudinal strain (GLS), the core parameters used to define cancer therapy-related cardiac dysfunction—as well as diastolic function, valvular heart disease, and pericardial structural abnormalities [34,35]. Cardiac magnetic resonance (CMR) offers superior accuracy and reproducibility in quantifying cardiac volumes and function, together with advanced tissue characterization (e.g., edema and fibrosis), serving as the gold standard when detailed structural assessment is required. However, its use is limited by cost, availability, and patient factors [34,35]. CMR is therefore recommended when echocardiographic windows are suboptimal or when tissue characterization is clinically relevant for risk stratification. Cardiac computed tomography is seldom performed in cancer survivors and is generally reserved for selected scenarios, such as the evaluation of coronary artery disease, pericardial pathology, or when other modalities are insufficient, with the caveat of radiation exposure and contrast administration [36].

The 2022 ESC/ICOS guidelines recommend monitoring with TTE and cardiac biomarkers at 3–6 and 12 months post-treatment completion for high-risk survivors, followed by TTE every 2 years in CCS and ACS until 5 years, and every 5 years thereafter [37]. Non-invasive coronary artery disease screening is recommended every 5 years in asymptomatic patients who received a mean heart dose > 15 Gy during radiotherapy [37,38].

Despite these structured recommendations, key evidence gaps persist. The optimal timing, modality, frequency, and cost-effectiveness of imaging remain uncertain, as does the role of circulating biomarkers and emerging digital health technologies in refining long-term monitoring. Addressing these gaps will be crucial for translating existing frameworks into measurable reductions in CVD burden among cancer survivors.

## 5. Impact of Exercise on Cardiometabolic Risk in Cancer Survivors

### 5.1. Exercise as a Cardiometabolic Intervention

Physical activity (PA), whether performed as structured physical exercise (e.g., strength or aerobic training) or as leisure-time recreational activity, confers wide-ranging benefits across multiple physiological systems, and constitutes a cornerstone intervention for individuals living beyond cancer [39,40]. Epidemiological evidence consistently links higher post-diagnosis PA levels with improved morbidity and survival outcomes for several diseases, including cancer [40,41]. For example, among breast cancer survivors, engagement in regular physical activity has been associated with reductions in all-cause mortality, cancer recurrence, and the incidence of cardiovascular events, including coronary artery disease and heart failure [34,36]. Beyond survival benefits, PA also alleviates cancer-related fatigue, promotes healthy weight management, strengthens immune regulation, and ultimately contributes to improved healthspan and quality of life [30,42,43].

Mechanistically, the broad physiological benefits of exercise are increasingly attributed to exerkines, bioactive molecules secreted in response to acute or chronic exercise that mediate systemic anti-inflammatory, metabolic, and cardiovascular adaptations via endocrine, paracrine, and autocrine signaling pathways [44]. Skeletal muscle acts as a secretory organ, producing a diverse array of exerkines, which coordinate inter-organ crosstalk and drive immune, metabolic, and cardiovascular adaptations [44]. Among the most studied exerkines are angiopoietin 1, fractalkine (CX3CL1), fibroblast growth factor 21 (FGF21), interleukins (IL-6, IL-8), musclin, myonectin, and vascular endothelial growth factor (VEGF), each contributing to the fine-tuning of immune responses, metabolic regulation, and vascular adaptation [45].

Recent evidence indicates that myokine expression in cancer survivors displays complex, context-dependent patterns, with FGF21 and musclin exerting nuanced effects on metabolic adaptation and muscle preservation. FGF21, a stress-inducible myokine involved in mitochondrial protection and lipid oxidation, appears dynamically regulated in cancer survivors, with exercise interventions significantly attenuating its proinflammatory overexpression [46]. Musclin, conversely, demonstrates protective mechanisms against muscle atrophy by restraining proteolysis and preserving muscle fiber area in preclinical cancer models [47]. Exposure to radiotherapy has been shown to dysregulate myokine signaling and thereby contribute to chronic tissue dysfunction [48]. Although differential exerkine patterns between adult and childhood survivors remain incompletely characterized, emerging studies suggest these molecules act as developmental-stage–dependent metabolic signals that may modulate early organ vulnerability [49]. Further targeted research is warranted to delineate myokine-specific pathways underlying cardiometabolic adaptation in CCS. For example, differential mRNA expression profiles in peripheral blood of anthracycline-exposed childhood cancer survivors with and without cardiomyopathy, and the modulation of these profiles following structured exercise interventions [50].

Importantly, these mediators are increasingly recognized for their capacity to counteract inflammation and mitigate cardiotoxicity—one of the most severe complications of cancer therapy [51]. Together, this molecular signaling framework provides a compelling rationale for positioning exercise not merely as supportive care, but as a potential disease-modifying strategy in cancer survivors.

### 5.2. Exercise and Cardiotoxicity Mitigation

Cancer therapies, particularly anthracyclines, are well known to induce cardiotoxic damage and accelerate the development of cardiovascular disease [51,52]. Exercise has emerged as a promising therapeutic strategy for both preventing and attenuating cardiotoxicity [52,53].

A recent meta-analysis reported that structured exercise improves cardiorespiratory fitness and peak oxygen consumption (VO_2_ peak) in patients exposed to cardiotoxic agents, although its ability to counteract chemotherapy-induced cardiac dysfunction directly remains uncertain [53].

The mechanistic rationale for exercise-induced cardioprotection is compelling; however, high-quality randomized controlled trials specifically targeting childhood and adolescent cancer survivors remain scarce. Most existing evidence derives from small adult cohorts or mixed-age samples with substantial heterogeneity in treatment exposures, exercise modalities, and outcome definitions. Nevertheless, preliminary studies indicate that exercise can exert cardioprotective effects in childhood cancer survivors through multiple molecular and physiological pathways. Mechanistically, exercise modulates gene expression and stimulates cardiovascular adaptations that counteract treatment-induced injury [54]. Aerobic training interventions have consistently demonstrated improvements in cardiorespiratory fitness and functional capacity in this population [55,56]. Moreover, structured exercise appears to preserve left ventricular function and attenuate treatment-related cardiac dysfunction [57]. Despite these encouraging findings, researchers consistently emphasize the need for larger, and methodologically robust randomized controlled trials to elucidate underlying molecular mechanisms, refine dosing parameters, and standardize exercise prescriptions in pediatric and adolescent cancer survivors [58]. Acknowledging this gap strengthens the rationale for translational research aimed at validating exercise as a therapeutic strategy capable of modulating cardiotoxicity and long-term cardiovascular risk in this vulnerable population.

Additionally, improvements in myocardial and vascular VEGF expression have also been observed [53]. Additional research is needed to elucidate the cardioprotective effects of PA more comprehensively, particularly on ischemic events, thrombotic complications, and inflammatory reactions in chemotherapy-exposed cardiovascular tissue [51,52]. Notably, the current evidence regarding the harms of physical exercise in this population remains of very low certainty due to data scarcity, poor reporting, and high risk of bias in studies [53]. This highlights the urgent need for high-quality randomized trials to inform and strengthen clinical recommendations.

### 5.3. Guideline-Based Approaches to Exercise Prescription

Current guidelines recommend a combined approach of aerobic and strength training (also known as resistance training) as the most effective method to reduce cardiometabolic risk and optimize quality of life in cancer survivors [43,59]. For specific populations, such as patients with lung cancer, preoperative combinations of respiratory exercises and aerobic training have shown positive outcomes [59]. A comprehensive pre-exercise clinical evaluation is crucial for individuals with comorbidities, such as peripheral neuropathy, arthritis, compromised bone health, lymphedema, or bone metastases [60]. When tailored to patient needs, exercise interventions are generally considered safe and well-tolerated [60]. The American Heart Association advocates for a four-step referral algorithm for Cardio-Oncology Rehabilitation (CORE), emphasizing that initiation can occur at any stage of cancer treatment by any healthcare professional and should involve a collaborative, multidisciplinary team approach [61,62]. In alignment with this, the ICOS-CORE 2025 statement underscores exercise as a core component of survivorship care, recommending personalized, risk-stratified training programs to mitigate cardiometabolic burden, enhance functional recovery, and improve long-term outcomes [63].

Furthermore, exercise modulates immune function, offering potential benefits in the prevention of inflammation-driven pathologies [64]. Preclinical evidence also supports exercise-induced suppression of tumorigenesis, likely mediated through reprogramming of metabolic and immune dysregulation, although the precise molecular pathways remain under investigation [65,66]. Collectively, these findings underscore exercise as a disease-modifying strategy in cancer survivors, capable of optimizing cardiometabolic health, functional capacity, and overall longevity. However, the molecular pathways and mediators that induced metabolic and immune benefits of controlled exercise in cancer patients and eventually cancer survivors are under ongoing investigation.

## 6. Molecular Pathways Linking Low-Grade Inflammation and Cardiometabolic Adaptation to Exercise in Cancer Survivors

Low-grade inflammation is characterized by an increase in proinflammatory cytokines, including IL-6 and TNF-α, as well as acute-phase proteins such as C-reactive protein (CRP) [67]. In cancer survivors, this chronic subclinical inflammatory state may result directly from specific antineoplastic therapies, particularly anthracyclines and radiotherapy, which induce a proinflammatory microenvironment, accelerating atherogenesis and impairing long-term cardiometabolic function [67]. Randomized controlled trials have demonstrated that regular aerobic exercise significantly reduces CRP levels by approximately 30–32% after approximately 10 weeks of training [68]. A recent meta-analysis confirmed significant reductions in both CRP and IL-6 with exercise programs in middle-aged and older adults [69]. For instance, in older adults (≥65 years), physical training was associated with modest but significant reductions in IL-6, TNF-α, and CRP compared with sedentary controls [69]. In the context of breast cancer survivors, exercise-mediated reductions in proinflammatory biomarkers such as IL-6, TNF-α, and CRP have been linked with lower mortality, reduced recurrence risk, and lower hospitalization rates [70]. A meta-analysis by Abbasi et al. (2022), conducted in women with breast cancer, found that exercise interventions significantly reduced CRP levels. Although average effects on IL-6 and TNF-α did not reach statistical significance, this meta-analysis suggests a favorable impact of exercise training on clinically relevant outcomes related to cancer patients, including mortality, survival rates, and the ability to perform activities of daily living [71].

With respect to the pleiotropic role of exerkines, IL-6 functions as a double-edged cytokine. In pathophysiological metabolic contexts, it acts as a proinflammatory molecule; however, during acute exercise, it is secreted by skeletal muscle as a myokine with anti-inflammatory properties, stimulating the release of IL-10 and IL-1ra while inhibiting TNF-α production [72]. Experimental studies have demonstrated that physical exercise, as well as infusions of recombinant IL-6 within physiological ranges, completely attenuates endotoxin-induced TNF-α elevation, supporting the notion that muscle-derived IL-6 mediates the anti-inflammatory effect of exercise [73]. Indeed, a meta-analysis indicated that exercise in breast cancer survivors, as well as in healthy populations, significantly reduces IL-6 (weighted mean difference ≈ −0.55 pg/mL). The best results are obtained with longer-duration programs (>11 weeks) and sessions lasting > 45 min [74]. Additionally, significant decreases in TNF-α have been observed in physically active breast cancer survivors, possibly linked to concomitant reductions in fat mass and diminished activation of adipose tissue–associated inflammatory pathways [75].

Additional mechanisms at the metabolism-inflammation interface are also relevant to exercise response. Adipokines produced by adipose tissue—particularly adiponectin—possess anti-inflammatory and antitumor properties. In overweight populations and breast cancer patients, regular exercise has been shown to increase adiponectin and reduce leptin levels, contributing to a less inflammatory metabolic environment [76]. This effect is particularly relevant because low adiponectin and elevated leptin, both characteristic of obesity, promote chronic low-grade inflammation and are linked to worse cancer prognosis. Thus, by partially reversing this adipocytokine profile, physical activity may attenuate the systemic proinflammatory state associated with obesity and cancer. In addition, exercise reprograms the immunometabolic system, reducing the expression of proinflammatory mediators (such as CRP, TNF-α, and IL-6) and favoring a sustained anti-inflammatory profile [77]. 

These molecular and immunometabolic mechanisms underpin the rationale for cardio-onco-rehabilitation, conceived as an integral therapeutic model for cancer survivors. Evidence in breast cancer is particularly robust: combined exercise programs (aerobic + resistance) show more robust anti-inflammatory responses than single-modality interventions [76]. Moreover, meta-regressions analyses have identified a dose–response relationship between intervention duration/intensity and IL-6 changes: interventions > 11–12 weeks and sessions > 45 min consistently yield more substantial IL-6 reductions [78]. Similarly, high-intensity exercise emerges as an effective stimulus for activating beneficial molecular pathways that affect both inflammation and metabolism, consolidating its role as a high-impact preventive strategy for cardiometabolic complications in populations with chronic inflammation [79]. In this context, the implementation of supervised aerobic and resistance exercise protocols of moderate to vigorous intensity, performed regularly for periods longer than ~12 weeks, has emerged as the most effective non-pharmacological intervention to modulate inflammatory biomarkers in cancer survivors [71] stably. These protocols not only reduce proinflammatory cytokines (IL-6, TNF-α, CRP) while increasing anti-inflammatory mediators (IL-10, adiponectin), but also improve key clinical outcomes such as cardiorespiratory fitness, body composition, and patient-reported quality of life [80].

In conclusion, physical exercise constitutes a cornerstone for both primary prevention (reducing the risk of incident CV disease) and secondary prevention (mitigating risk in individuals with existing cardiovascular disease) due to its pleiotropic systemic effects. The mechanisms underlying these benefits span multiple physiological levels, including mitochondrial dynamics, vascular reactivity, and autonomic homeostasis [81]. Among these, the inhibition or attenuation of proinflammatory pathways mediated by exercise-induced molecules (exerkines) appears to be the central mechanism driving both short- and long-term health improvements. Although the concept of “exercise in pills” currently seems unrealistic, decades of research in this area have yielded valuable insights, particularly in identifying novel molecular targets for cardiometabolic protection and therapeutic intervention.

## 7. Neutrophil Extracellular Traps and Their Potential Role in Muscle Metabolism and Exercise Intensity

*Neutrophil extracellular trap* (NET)-osis is defined as a neutrophil death pathway characterized by the disassembly of the nucleus and the release of chromatin and modified proteins, forming structures called NETs that trap microorganisms and promote inflammation [82]. This multistage process involves membrane disintegration and chromatin decondensation, ultimately leading to plasma membrane rupture and NET release. These events could trigger various inflammatory pathways, cytokines and infectious processes against microorganism infection [83]. However, NET release can also occur during pathological sterile inflammatory conditions, such as in atherosclerosis, stroke, and abdominal aortic aneurysm [84].

The role of NETs in skeletal muscle remains poorly understood, but theoretical emerging evidence suggests both harmful and adaptive effects depending on exercise intensity. At low-intensity exercise, the stimuli and magnitude of increases in some myokines may play a protective role in muscle and endothelial dysfunction in animal models [85,86]. However, in humans—for example, in obese patients—the results at low-intensity training determine a minimal impact on cardiometabolic risk factors respect to high and moderate-intensity [87]. On the other hand, at high-intensity exercise, induced pro-oxidant effects facilitated NET formation via elevating the NADPH oxidase-generated ROS in blood leucocyte of human samples [88]. Recent studies also show that baseline serum from older men induce greater baseline NETosis than younger men, and that 12 weeks of high-intensity interval training significantly reduced the induction of NETosis in older men [89]. This type of training involves repeated bouts of relatively intense exercise with alternating short recovery periods, and has shown beneficial effects on health parameters during aging and disease, including cancer [90,91]. However, there is no definitive clinical evidence to support a positive or negative effect of modulating NETosis in skeletal muscle with controlled physical activity or high- or low-intensity training. In this case, mechanistic studies directly linking them are lacking.

Neutrophils play a crucial role in muscle regeneration after injury or intense exercise. Following damage, they are rapidly recruited to the injury site, where they release proinflammatory molecules and proteolytic enzymes that clear cellular debris and damaged tissue. Moreover, neutrophils can influence inflammatory resolution and muscle adaptation to different exercise intensities [92].

Beyond their well-characterized roles, neutrophils can also release NETs, which are DNA structures that can capture and eliminate pathogens. This process involves the release and transfer of cellular contents (e.g., DNA, histones, and granule proteins) from neutrophils to targeted cells. Once neutrophils expel the NETs, NETs can be found in plasma as circulating cell-free double-stranded DNA (cfDNA) [93]. 

For instance, NETs formation has been associated with skeletal muscle fibrosis following ischemia–reperfusion (IR) injury. Edwards et al. reported extensive NET formation and neutrophil infiltration in skeletal muscle after IR, and that inhibition of protein-arginine deiminase 4 (PAD4) can reduce NET formation and subsequent fibrosis with no impact on muscle regeneration [94]. Similarly, in another model of ischemia/reperfusion, DNase I treatment reduced NET detection in skeletal muscle after ischemia injury, although the alterations in muscle fiber structure and proinflammatory markers did not change significantly [95]. Along the same lines, other studies show that batroxobin, a defibrinogenating agent derived from viper venom, inhibits NETs formation, fibrinogen deposition, and subsequent tissue damage in ischemic skeletal muscle [96].

Additionally, neutrophil dysregulation has also been observed in idiopathic inflammatory myopathies. In this pathogenic context, some studies have described that NETs can interfere with myotube viability in a citrullinated histone-dependent manner, enhancing myotube death after 1 h of exposure, while no disruption was observed in myoblast proliferation. These findings support a pathogenic contribution of NETs to muscle damage and dysfunction [97].

On the other hand, exercise itself appears to regulate NET dynamics. Exercise intensity influences the magnitude of cfDNA release from neutrophils, suggesting that exercise can regulate NET formation and that neutrophils can release NETs in response to exercise [98]. Thus, exercise intensity and training status modulate cfDNA release and cytokine responses, contributing to the anti-inflammatory effects of regular exercise. Interestingly, Irisin, a myokine linked to the exercise response, has been found to reduce NETs’ formation via regulation of the p38/MAPK pathway in a mouse model of acute pancreatitis [99]. This highlights the potential therapeutic benefits of targeting NET formation in the prevention and treatment of muscle diseases. Based on the above, we suggest that NETs’ formation may exert both beneficial and detrimental effects on skeletal muscle. Understanding the underlying mechanisms of NET formation and its role in muscle physiology may have important implications for the development of therapeutic strategies to improve muscle recovery and prevent injury [100]. Moreover, given that the immune response is critical for recovery from muscle damage, the potential role of NETosis in resistance exercise, including the modulation of exerkines and circulating factors, could be essential in mediating muscle recovery [101].

Recent findings also suggest that NETosis may contribute to the cardiotoxicity of anthracyclines demonstrated that lymphoma patients treated with doxorubicin exhibit elevated circulating levels of NET DNA, which correlated with declines in LVEF [102]. Furthermore, in animal models, doxorubicin induces myocardial NET formation via High Mobility Group Box 1 (HMGB1) release, which interacts with Toll-Like Receptor 4 (TLR4) receptors in cardiomyocytes, promoting oxidative stress and ferroptotic cell death, culminating in cardiac remodeling and dysfunction. Inhibition of NETosis or HMGB1 neutralization attenuated these effects, confirming the causal link between NETs and anthracycline cardiotoxicity [103]. This suggests a plausible mechanism by which NETs promote local inflammation, cell death, and possibly residual fibrosis in cardiac tissue. Regarding exercise, although there are no specific studies in cancer survivors with cardiotoxicity, there is evidence in general contexts that intense or prolonged exercise modulates the body’s ability to induce or degrade NETs: some workouts increase the activity of nucleases (DNases) that degrade extracellular DNA, reducing cfDNA levels and decreasing the propensity of neutrophils to form NETs [89,104]. Taken together, this suggests that in cancer survivors, guided exercise could act by regulating the “NETotic balance”—limiting excessive NET formation and promoting their elimination—as a protective mechanism against the progression of post-chemotherapy cardiac damage.

**Figure 2:**
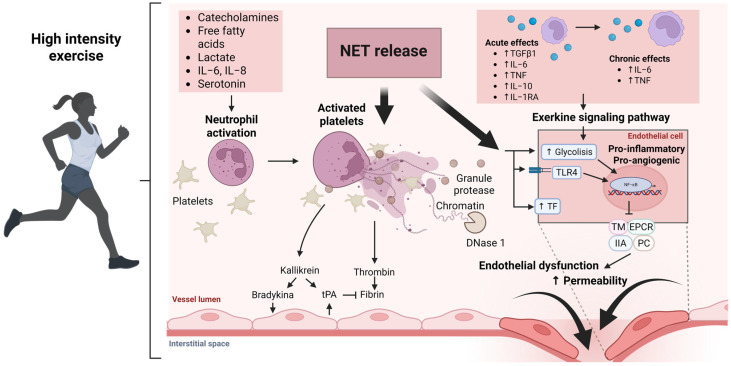
Molecular and cellular mechanisms induced by high-intensity exercise and modulation of neutrophil extracellular traps (NETs). Molecular and cellular mechanisms induced by high-intensity exercise and modulation of neutrophil extracellular traps (NETs). High-intensity exercise activates neutrophils and platelets through hemodynamic stress, catecholamines, and metabolic mediators such as lactate and exerkines (IL-6, IL-8, TNF-α). This leads to transient NET release and local proinflammatory signaling that modulates endothelial function via TLR4-dependent pathways, promoting glycolytic and angiogenic responses. In parallel, fibrinolytic activity (tPA) and DNase I-mediated NET degradation counteract vascular inflammation and permeability increases. The outcome depends on the dynamic balance between NET formation, clearance, and endothelial integrity. The arrows show a trombin and kalikrein activation; and pathways to modulate endothelial dysfunction. Abbreviations: DNase I, deoxyribonuclease I; EPCR, endothelial protein C receptor; IL, interleukin; IL-6, interleukin-6; IL-8, interleukin-8; IIA, coagulation factor IIa (thrombin); NETs, neutrophil extracellular traps; NFκB, nuclear factor kappa B; PC, protein C; TF, tissue factor; TFPI, tissue factor pathway inhibitor; TGFβ1, transforming growth factor beta 1; TLR4, Toll-like receptor 4; TM, thrombomodulin; TNF, tumor necrosis factor; tPA, tissue plasminogen activator; vWF, von Willebrand factor.

Regular physical activity is hypothesized to enhance the activity of nucleases (such as DNase I) that degrade extracellular DNA, thereby accelerating NET clearance. Consistent with this, an 8-month endurance exercise program in individuals with cardiovascular risk factors was shown to increase serum DNase activity while concomitantly decreasing circulating cell-free DNA levels. Such changes reduce the available DNA scaffold for NET formation and are associated with a lower propensity for neutrophils to undergo NETosis [104]. Similarly, high-intensity interval training (HIIT) in older adults significantly diminished the ability of their plasma/serum to induce NET formation, indicating that exercise can directly blunt excessive NETosis. By limiting aberrant NET release and promoting NET degradation, exercise may help alleviate NET-driven inflammation and tissue injury in the heart [89]. Thus, even though direct studies in cancer survivors are lacking, these findings suggest that structured exercise interventions could maintain a healthier “NETotic” balance and protect against ongoing anthracycline-induced cardiac damage (Figure 2).

## 8. An Integrated Model of Exercise-Based Intervention Against Cardio- and Immuno-Metabolic Risk in Cancer Survivors

Our group proposes a novel integrative framework for clinical follow-up, linking chemotherapy-induced cardiotoxicity in childhood cancer survivors with the protective and restorative effects of exercise from a molecular, physiological, clinical, and public health perspectives. This framework incorporates the concept of “cardio-onco-rehabilitation”, as an integrative model that combines clinical medicine (patient follow-up), exercise science (improving physical capacity), and molecular biology (cardiometabolic protection and anti-remodeling mechanisms). 

The interdisciplinary structure of this model integrates the following domains:

- Pediatric Oncology: survivorship follow-up.
- Cardiology: cardiovascular risk assessment, monitoring of chemotherapy-induced cardiotoxicity (TTE and cardiac biomarker surveillance).
- Exercise Physiology: individualized aerobic and resistance exercise protocols, and CRF assessment.
- Immunology/Inflammation: profiling of inflammatory markers and exerkines.
- Public Health: evaluation of both short- and long-term follow-up policies in Latin America and Chile (e.g., PINDA program).

**Figure 3:**
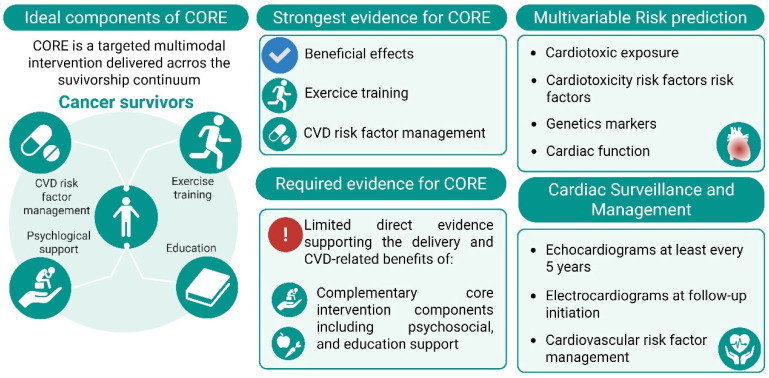
Conceptual CORE (Cardio-Oncology Rehabilitation) model illustrating key components of a multidisciplinary cardiometabolic rehabilitation framework for application in Latin America and Chile. The model integrates evidence-based strategies (ESC/ICOS guidelines), and proposes a multivariable risk-prediction approach to guide cardiac surveillance in Chilean cancer survivors (CS)—abbreviations: CVD, cardiovascular disease.

This transdisciplinary approach is based on the generation of new mechanistic and clinical evidence (Figure 3). The projection of this paradigm not only seeks to understand the mechanisms underlying exercise-induced cardioprotection against cardiotoxicity, but also supports the development of integrated intervention models combining clinical care (hospital-based protocols), personalized physical training regimens (supervised by physical therapists and exercise physiologist), and translational biomarkers to predict cardiovascular risk and exercise responsiveness. This type of approach can be further strengthened through the incorporation of biochemical markers (exerkines) as part of risk algorithms. This evidence could be groundbreaking in Latin America.

The expected outcomes of this paradigm include:

- The development of evidence-informed guidelines for oncologists and cardiologists on surveillance and exercise prescription.
- The integration of exercise programs into national health systems as a validated therapeutic strategy for cancer survivors.
- The identification of novel molecular targets for cardiometabolic and immunomodulatory therapies.

Ultimately, this model envisions exercise not merely as supportive care, but as a disease-modifying intervention with profound implications for longevity, quality of life, and resilience in cancer survivors.

## 9. Conclusions

We expect that optimizing cardiovascular risk by controlling cardiovascular risk factors, implementing structured physical training, and other multitask interventions will reduce the proinflammatory and adverse cardiometabolic profile associated with long-term CV risk in CS. NETosis (as a cell death type) research is a potential pathway that can plausibly be used to explore clinical approaches. Findings may identify novel pharmacological and exercise-responsive targets and could support health-policy measures to prevent medium-term cardiovascular complications in this growing survivor population.

## Data Availability

No new data was created and these data are unavailable due to privacy or ethical restrictions, derived from Local ethical Committee, East Health Services.

## Abbreviations

APC	Activated Protein C
AUC	Area Under the Curve
CCS	Childhood Cancer Survivors
cfDNA	Cell-Free DNA
CMR	Cardiac Magnetic Resonance
CORE	Cardio-Oncology Rehabilitation and Exercise
CRF	Cardiorespiratory Fitness
CRP	C-Reactive Protein
CS	Cancer Survivors (general)
CVD	Cardiovascular Disease
CX3CL1	Fractalkine
DNase I	Deoxyribonuclease I
DNA	Deoxyribonucleic Acid
ESC/ICOS	European Society of Cardiology/International Cardio-Oncology Society
FGF21	Fibroblast Growth Factor 21
HMGB1	High Mobility Group Box 1
HFA	Heart Failure Association
ICOS-CORE	International Cardio-Oncology Society—Cardio-Oncology Rehabilitation and Exercise
IL	Interleukin
IL-6	Interleukin-6
IL-8	Interleukin-8
IL-10	Interleukin-10
IL-1ra	Interleukin-1 receptor antagonist
IR	Ischemia–Reperfusion
LV	Left Ventricle/Left Ventricular
MAPK	Mitogen-Activated Protein Kinase
MPO-DNA	Myeloperoxidase–DNA complex
NCDs	Non-Communicable Diseases
NETs	Neutrophil Extracellular Traps
NETosis	Neutrophil Extracellular Trap Formation
PA	Physical Activity
PAD4	protein-arginine deiminase 4
SASP	Senescence-Associated Secretory Phenotype
TF	Tissue Factor
TFPI	Tissue Factor Pathway Inhibitor
TLR4	Toll-Like Receptor 4
TNF-α	Tumor Necrosis Factor-alpha
TM	Thrombomodulin
TTE	Transthoracic Echocardiography
VEGF	Vascular Endothelial Growth Factor
VO_2_ peak	Peak Oxygen Consumption
vWF	von Willebrand Factor
YAP	Yes-Associated Protein
